# Genetic Variability and Population Structure of Ethiopian Yams (*Dioscorea* spp.) Based on SSR Markers

**DOI:** 10.1002/ece3.71562

**Published:** 2025-06-05

**Authors:** Gerezgiher Mekonen Gebrhud, Hewan Demissie Degu, Eleni Shiferaw Tessema, Tsegaye Babege Worojie

**Affiliations:** ^1^ Ethiopian Biodiversity Institute Addis Ababa Ethiopia; ^2^ School of Plant and Horticulture Science Hawassa University Hawassa Ethiopia; ^3^ Department of Horticulture Mizan‐Tepi University Mizan‐Teferi Ethiopia

**Keywords:** *Dioscorea* spp., genetic variability, population structure, SSR markers, yam

## Abstract

In order to implement breeding and conservation measures, it is crucial to evaluate the extent of genetic diversity. Hence, this study aimed to evaluate the genetic diversity and population structure of 31 yam accessions collected from various agro‐ecologies in Ethiopia based on 10 SSR markers. Our results showed the existence of a wide range of genetic diversity among the yam accessions. A total of 72 alleles were amplified and the number of alleles per marker ranged from 5 to 11, with an average of 7.2. The average values of expected and observed heterozygosity were 0.38 and 0.30, respectively. The average polymorphic information content per marker was found to be 0.30, indicating the markers are reasonably informative towards detecting the genetic diversity among accessions. The level of population polymorphism was found to vary from 68.06% (Sheka) to 97.22% (Bench‐Maji and Gambella), with an average of 85.2% among populations. Accessions from Bench‐Maji and Gambella resulted in higher values for all diversity indices; hence, this area can thus be considered a potential site for in situ conservation. Analysis of molecular variance showed that the within‐population variations contributed more to the observed genetic diversity. Similar results were also evident in the multivariate analyses, in which accessions were grouped without a distinct pattern of geographic origins. The high within‐population variation indicated the potentiation of on‐farm diversity for establishing a breeding and conservation program.

## Graphical abstract

The result showed that the SSR markers used in this study were efficient and informative for genetic diversity study of yams. The pattern of diversity across the collection sites showed that Bench‐Maji and Gambella populations resulted in higher values for the genetic diversity parameters assessed, and these areas could thus be considered as a potential conservation site for diverse yam species.

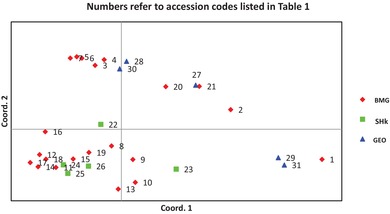

## 1 Introduction

The term ‘yam’ is only used to describe members of the genus *Dioscorea* that belong to the family Dioscoreaceae. The genus *Dioscorea*, which yam is a part of, has around 600 species (Burkill 1960). However, only a few of them are in cultivation. Several yam species may have originated in Ethiopia and are among the crops with wild relatives in the country (Harlan 1969; Dadi and Engels 1982; Edwards 1991; Engels and Hawkes 1991; Miege and Demissew 1997). These reports contributed a lot towards exploring *Dioscorea* species of Ethiopia and their spatial patterning in the country. Though similar reports are available in more general references (Westphal 1975; Engels et al. 1991), much of these reports listed a few of the *Dioscorea* species available in Ethiopia. Besides, only limited research works address the issue of the within species diversity in Ethiopian yams.

For inferring the genetic diversity in yam, several studies have been conducted globally. These include a record and characterization of yam landraces using morphological traits (Tamiru et al. 2008, 2011; Loko et al. 2015; Asfaw et al. 2021), molecular markers (Tamiru et al. 2007; Tostain et al. 2007; Mengesha et al. 2013a, 2013b; Loko et al. 2017) and a combination of morphological and molecular markers (Darkwa et al. 2020; Agre et al. 2021; Cao et al. 2021; Gabriel et al. 2021). Until recently, much of our knowledge of yam diversity comes from the yam belt of West Africa. Ethiopia is located outside the yam belt of West Africa and is regarded as an isolated center of yam cultivation (Norman et al. 1995). Yam genetic diversity may be extensive in Ethiopia, but it is not put to effective use, as the available diversity is poorly known. The yam diversity available in the country is thus needed to be studied in detail.

Previous studies in Ethiopia showed the occurrence of an extensive number of farmer's varieties with a varying degree of spatial variation (Hildebrand 2003; Tamiru et al. 2008; Worojie et al. 2021a, 2021b). Most of these are distinct, and the general structure of morphological diversity was mostly consistent with the local classification system (Tamiru et al. 2011; Asfaw et al. 2021). Yet, some landraces that farmers considered distinct have been grouped together and did not exhibit any morphological variations. On the other hand, morphological differences were found among some landraces with similar vernacular names. This provides a clue to the importance of additional explorations and, more notably, DNA‐based studies involving high resolution markers to unravel these difficulties.

Diversity analysis of yam genotypes at the molecular level has been done in yam collections from different parts of Ethiopia (Tamiru et al. 2007; Mengesha et al. 2013a, 2013b; Bekele 2014; Mulualem et al. 2018; Atnafua and Endashaw 2021). These studies have played a significant role in terms of identifying the level of genetic diversity within the yam germplasm. But the amount of variation at the genetic level and population differentiation was not consistent in these studies. Some of these studies considered only cultivated yams (Tamiru et al. 2007) while others used samples representing a specific growing region (Mulualem et al. 2018). The use of molecular markers involving a wide‐ranging account of yam accessions is expected to contribute towards a detailed analysis of genetic diversity within yam accessions. The insight that such analysis provides is important for yam improvement and its conservation in Ethiopia. Thus, this study aims to investigate the genetic diversity as well as the population structure of some yam landraces from Ethiopia using SSR markers.

## 2 Materials and Methods

### 2.1 Study Population and Study Area

Initially, young leaf specimens were collected from a total of 136 accessions during the period from March to May 2020 and dried in plastic ziplock bags with silica gel. Of these, 61 accessions were collected from farmers' fields in Bench‐Maji and Sheka Zones of southwestern Ethiopia, while the rest were collected from the field gene bank conserved ex situ at Choche. Despite the fact that 136 accessions were subjected to amplification, 105 of them have failed to show better amplification products. For this reason, we managed to consider 31 accessions (23 *D. cayenensis* complex, 6 *D. alata* and 2 *D. bulbifera* ) in this study. Out of the 31 accessions, 23 (i.e., all the *D. cayenensis* complex accessions) represented those that were collected in farmers' fields from Southwest Ethiopia, while the rest were those that were collected from the field gene bank.

The accessions considered in this study represented Bench‐Maji, Sheka, Gambella, Gedio, and Oromia floristic regions. Therefore, three populations were identified based on adjacent floristic regions, with Bench‐Maji and Gambella (BMG), Sheka (SHK) and Gedio and Oromia (GEO) populations for Bench‐Maji and Gambella, Sheka, and Gedio and Oromia floristic regions, respectively. Supporting Information S1 (ESM 1) presents the lists of local and scientific names together with the geographic coordinates where these accessions were collected. ESM 2 also presents a map, indicating the geographic origin of accessions considered in this study.

### 2.2 DNA Isolation and Quantification

Genomic DNA was extracted from 31 samples using the Cetyl Tri‐methyl Ammonium Bromide (CTAB) procedure modified from Keim et al. (1989) at the Molecular Laboratory of the Ethiopian Biodiversity Institute, Addis Ababa, Ethiopia. Leaf tissue (100 mg) was ground into powder in sterile mortars and pestles and transferred to 2 mL microcentrifuge tubes. Extraction buffer (700 μL) containing 1 M Tris HCl (pH 8.0), 5 M NaCl, 0.5 M EDTA, 1% CTAB, and 0.4% Mercaptoethanol was added to each tube, and the tubes were incubated at 65°C in a water bath for 90 min, 300 μL chloroform/Isoamyl alcohol (24:1) was added to 400 μL supernatant taken from each tube to remove the protein contaminants. The concentration and purity of the DNA samples were checked using a Nano‐Drop Spectrophotometer at the Plant Genetics Research Laboratory, Addis Ababa University, Ethiopia. DNA quality was checked using 0.8% agarose gel in 0.5X TBE buffer.

### 2.3 PCR Amplification

SSR markers previously identified for different *Dioscorea* species by Tostain et al. (2006) were assessed. Out of 12 SSR markers, only 10 showed polymorphism, producing well‐defined and reproducible alleles and thus were selected for use in this study (Table 1). The optimal conditions were identified after performing PCR optimization for all primer pairs. After identifying the optimum PCR conditions of the primer pairs, PCR was carried out in a final volume of 15 μL, consisting of 5 units of *Taq* DNA polymerase, 10X amplification buffer, 25 mM of MgCl_2_, 10 μM of each primer, 2.5 μM of each dNTP, and 30 ng/μl of DNA template.

**TABLE 1:** Characteristics of the 10 SSR markers used in the analysis of yam accessions.

Markers	Primer sequences (5′ to 3′)	Repeat motifs	Allele size (bp)		Annealing temperature (°C)
			Expected	Observed	
Ya1	F: TATAATTCGGCCAGAGG R: TGTTGGAAGCATAGAGAA	(AG)_18_	113–254	200–308	48.5
Ya2	F: CCCATGCTTGTAGTTGT R: TGCTCACCTCTTTACTTG	(AG)_6_	190	189–264	50.5
Ya3	F: TGTAAGATGCCCACATT R: TCTCAGGCTTCAGGG	(CT)_9_	174	172–208	45
Ya5	F: TTGAACCTTGACTTTGGT R: GAGTTCCTGTCCTTGGT	(CT)_18_	152	157–239	50.5
Ya6	F: AACATATAAAGAGAGATCA R: ATAACCCTTAACTCCA	(GAA)_5_	123–180	124–200	45.5
Ya7	F: CATCAATCTTTCTCTGCTT R: CCATCACACAATCCATC	(GT)_8_	127–170	111–205	50.5
Ya8	F: AGACTCTTGCTCATGT R: GCCTTGTTACTTTATTC	(AG)_15_	130–190	105–215	50.5
Ya9	F: TCCCCATAGAAACAAAGT R: TCAAGCAAGAGAAGGTG	(GA)_8_	103–221	165–245	51
Ya10	F: TTCCCTAATTGTTCCTCTTGTTG R: GTCCTCGTTTTCCCTCTGTGT	(AG)_12_	104–183	310–606	60
Ya12	F: AATGCTTCGTAATCCAAC R: CTATAAGGAATTGGTGCC	(TG)_6_	152–198	189–221	50.5

Abbreviations: *F*, forward primer sequence; *R*, reverse primer sequence.

The Hybaid PCR express thermal cycler (Hybaid, UK) was used to perform PCR under the following amplification conditions. The primer is denatured at 94°C for 5 min and then repeated 35 times, with 30 s of denaturation at 94°C and 30 s at the designated annealing temperature for each primer and 45 s at 72°C, with a final extension at 72°C for 10 min (Tostain et al. 2006). The amplification products were electrophoresed in 6% non‐denaturing polyacrylamide gels for 30 min at 100 V and then at 120 V for 2 h. The gel was treated with 500 mL fix solution (0.5% glacial acetic acid and 10.5% Ethanol) for 25 min before the staining procedure. A digital camera was used to photograph gels stained using a silver staining procedure (Huang et al. 2018).

### 2.4 Data Analysis

Band sizes were scored with UVITEC version 16.09b software and exported to Excel. A binary matrix was created to record the presence (1) or absence (0) of alleles for each microsatellite locus per accession, using the scored genotypic data. Power Marker ver. 3.25 (Liu and Muse 2005) was used to determine locus‐based diversity indices: major allele frequency (MAF), gene diversity (GD), and polymorphic information content (PIC) The number of effective alleles (Ne), expected heterozygosity (He), Shannon's Information Index (I) and percentage of polymorphic loci (PPL) over the sub‐populations and the entire loci, Nei's standard genetic distance, and Population Pairwise Nei's standard genetic distance, Analysis of Molecular Variance (AMOVA), population differentiation (PhiPT), and Principal Component Analysis (PCoA) were computed using GenAlEx ver. 6.501 (Peakall and Smouse 2012).

Cluster analysis was carried out using DARwin software version 6.0 (Perrier and Jacquemoud‐Collet 2006). A dendrogram was generated based on the dissimilarity matrix as input data in order to visualize the patterns of accession clustering. The Unweighted Neighbor Joining (NJ) method (Saitou and Nei 1987) was constructed using Power Marker ver. 3.25 (Liu and Muse 2005) and viewed using Mega 6 (Kumar et al. 2018). A Bayesian clustering analysis was conducted using STRUCTURE version 2.3.4 (Pritchard et al. 2000; Falush et al. 2003). In this analysis, individuals are probabilistically assigned to one of the predefined K populations to identify the optimal number of genetic groups (Evanno et al. 2005). In order to ascertain the most probable number of populations (K), a burn‐in period of 50,000 was implemented for each run and data were collected over 50 000 MCMC replications for K ranging from 1 to 10, using 20 iterations for each K. The most probable K value was determined using the maximum value of ∆K according to Evanno et al. (2005) based on a model allowing admixture with correlated allele frequencies without any prior information. The bar plot for the optimum K was determined using Clumpak beta version (Kopelman et al. 2015).

## 3 Results

### 3.1 Level of Marker Polymorphism and Overall Genetic Diversity

Locus based diversity indices are presented in Table 2. The 10 SSR markers had 72 amplified alleles, with an average of 7.2 alleles per locus. The number of alleles varied widely between the markers, ranging from 5 for (Ya3 and Ya7) to 11 for (Ya10). The mean number of effective alleles was 1.6, with a minimum of 1.39 and a maximum of 1.97 for loci Ya1 and Ya3, respectively. Allelic frequency data revealed that only 3 (4.2%) of the alleles were rare (alleles with frequency between 0.01 and 0.05) while the remaining alleles, 69 (95.8%) showed a frequency of > 0.05. Likewise, the major allele ranged from 0.60 (Ya6) to 0.84 (Ya1), with a mean value of 0.71. Gene diversity, or expected heterozygosity, was moderate, ranging from 0.23 for loci Ya1 to 0.48 for locus Ya6, with an average value of 0.38. The observed heterozygosity ranged from 0.15 (Ya1) to 0.45 (Ya3) with a mean value of 0.30. The 10 SSR markers showed a moderate polymorphic information content (PIC), ranging from 0.18 (Ya1) to 0.36 (Ya6) with an average value of 0.30.

**TABLE 2:** Polymorphism detected by 10 SSR markers in 31 yam landraces.

Marker	Na	Ne	MAF	GD/He	Ho	PIC
Ya1	7	1.39	0.84	0.23	0.15	0.18
Ya2	9	1.67	0.70	0.40	0.36	0.32
Ya3	5	1.97	0.72	0.40	0.45	0.31
Ya5	6	1.73	0.68	0.42	0.39	0.33
Ya6	6	1.75	0.60	0.48	0.39	0.36
Ya7	5	1.49	0.81	0.26	0.22	0.22
Ya8	8	1.43	0.68	0.41	0.26	0.33
Ya9	8	1.62	0.67	0.42	0.33	0.33
Ya10	11	1.45	0.70	0.38	0.17	0.30
Ya12	7	1.62	0.74	0.36	0.25	0.29
Mean	**7.2**	**1.61**	**0.71**	**0.38**	**0.30**	**0.30**

1 2

### 3.2 Genetic Diversity Among Populations

By pooling the data presented in Table 3, the genetic diversity of the three populations were evaluated. The results showed that Bench‐Maji and Gambella (BMG) populations showed the highest genetic variability (*N* = 70, Na = 7, Ne = 1.64, He = 0.34, I = 0.51, PPL = 97.22) (Table 3), all of which were higher than their respective grand means. The populations of Gedeo and Oromia (GEO) also showed high levels of the genetic diversity parameters, which were either equivalent to or higher than their respective grand means. For all practical purposes, the populations of Sheka (SHK) showed the lowest value for all the genetic diversity indices assessed. The BMG populations had five amplified private alleles, but the remaining populations did not have any amplified private alleles (Table 3).

**TABLE 3:** Genetic diversity among the three yam populations as estimated using 10 SSR markers.

Populations	Ns	N	Na	Ne	Ap	He	I	PPL (%)
Bench‐Maji and Gambella (BMG)	21	70	7	1.64	5	0.34	0.51	97.22
Sheka (SHK)	5	49	4.9	1.4	0	0.23	0.34	68.06
Gedeo and Oromia (GEO)	5	65	6.5	1.5	0	0.29	0.43	90.28
Total	**31**	**184**	**18.4**	**4.54**	**5**	**—**	**—**	**—**
Mean	**—**	**61.33**	**6.13**	**1.51**	**1.67**	**0.29**	**0.43**	**85.2**

*Note:* The bold values indicate the total and average values for each parameters per population.

Abbreviations: Ap, private alleles; He, expected heterozygosity; I, Shannon's information index; N, total number of alleles per population; Na, mean number of alleles per locus; Ne, number of effective alleles per locus; Ns, number of samples; PPL, percentage of polymorphic loci.

### 3.3 Analysis of Molecular Variance

The total genetic variation was divided into within and among populations by analysis of molecular variance (AMOVA), showing that 92% of the variation detected was found within populations while the variation among the collection sites contributed only 8%. The genetic differences between populations were moderate but significant (Table 4). The standard genetic distance between all possible pairs of yam genotypes by Nie's was between 0.069 and 0.75. The populations of BMG had most closely related genotypes, while the populations of GEO had the most distantly related genotypes. The Pairwise Nei's genetic distances between the populations ranged from 0.039 to 0.17. The pairwise genetic distance between SHK and GEO populations was observed to be the highest (1.17) with Fst = 0.28. On the other hand, the lowest pairwise genetic distance (0.039) was observed between BMG and SHK populations with Fst = 0.06 (Table 5).

**TABLE 4:** Analysis of molecular variance (AMOVA) among populations and within populations.

Sources of variation	DF	SS	MS	Estimated variation	%
Among populations	2	48.57	24.28	1.3	8
Within populations	28	399.2	14.26	14.26	92
Total	30	447.8		15.58	100
Stat	Value	*P* (rand ≥ data)			
PhiPT	0.085	0.044			

**TABLE 5:** Nei's standard genetic distance between populations (below diagonal) and population pairwise Fst (above diagonal) for the three populations studied.

Population	BMG	SHK	GEO
BMG	****^a^	0.06	0.18
SHK	0.039	****^a^	0.28
GEO	0.10	0.17	****^a^

^a^ Similar populations.

### 3.4 Cluster Analysis and Population Structure

**FIGURE 1:**
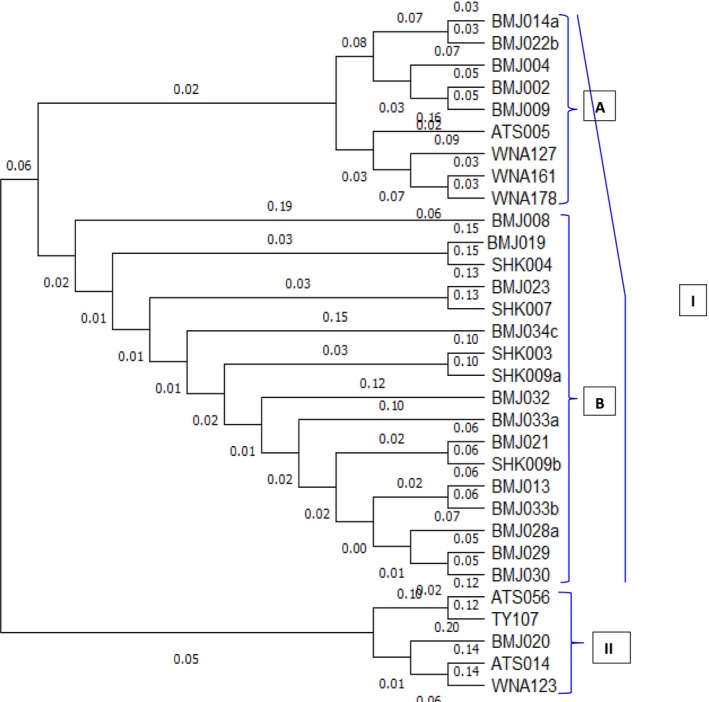
Unweighted Neighbor Joining (NJ) dendrogram showing genetic relationship of 31 yam accessions based on 10 SSR markers.

The cluster study based on Neighbor‐Joining (NJ) methods clustered the three populations into two major clusters, with each of which containing accessions from distinct geographic origin. The first major cluster constituted accessions from all geographic origins, while the second major cluster included accessions from BMG and GEO populations (Figure 1). Two sub‐clusters were formed within the first major cluster. The first sub‐cluster consisted of 9 accessions, with 6 (67%) and 3 (33%) accessions from BMG and GEO, respectively. The second sub‐cluster is composed of 17 accessions, where the majority of the accessions, 12 (71%) were from BMG while 5 (29%) were from SHK populations. Of the five accessions placed in cluster II, 3 (60%) were from BMG while 2 (40%) were from GEO populations (Figure 1). None of the accessions considered in this study form a duplicate.

**FIGURE 2:**
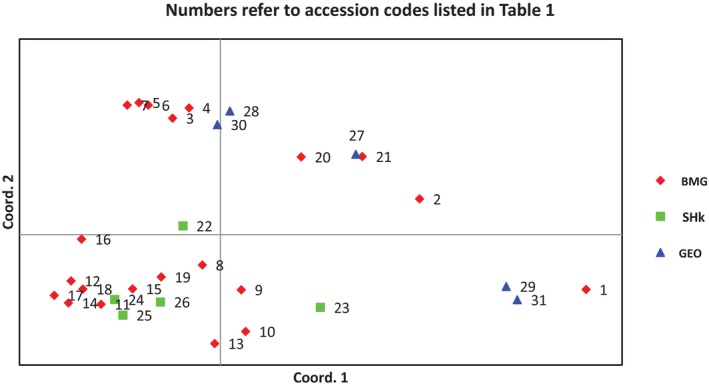
Scatter plots from the principal coordinate analysis based on 10 SSR markers for the 31 yam accessions corresponding to three distinct populations.

**FIGURE 3:**
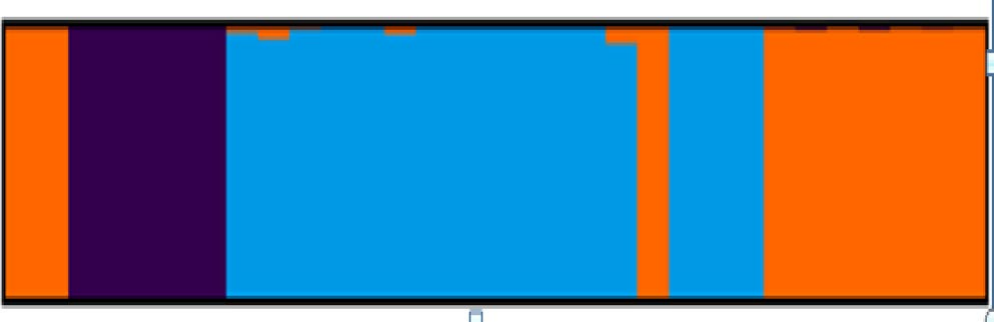
Estimated number of populations (K) derived from the structure clustering analyses. The magnitude of ∆K was calculated using the method described by Evanno et al. (2005).

The principal coordinate analysis (PCoA) showed a considerable variability among the 31 accessions, with the first three PCs together explained 42% of the total variation. In the two‐dimensional scatter plot, accessions with different geographic origins were grouped together (Figure 2). None of the populations formed a separate group in the scatter plot, and it thus agrees with the cluster study. This observation is also supported by STRUCTURE analysis using a model‐based Bayesian algorithm, which allowed identification of three clusters (*k* = 3) as being optimal to capture the major structure in the full data set (Figure 3). Based on this value, the population structure bar plot indicated the existence of a considerable admixture and hence showed no clear grouping of populations according to their geographic origins.

## 4 Discussion

### 4.1 | Level of Marker Polymorphism and Overall Genetic Diversity

Out of the 12 SSR markers used in this study, only 10 of them showed clear and reproducible fragments. Altogether72 alleles were produced by the 10 SSR markers, with varying effective number of alleles, number of alleles per locus, and major allelic frequency. The overall number of alleles per primer recorded in this study was relatively greater than the reports by Mengesha et al. (2013a) and Bekele (2014), who reported a total of 60 and 68 alleles using SSR markers, respectively. It was also much higher than the findings of Mulualem et al. (2018) and Siqueira et al. (2012), who reported a total of 30 and 45 alleles on yam accessions from Ethiopia and Brazil, respectively. Nonetheless, the total number of alleles per primer recorded in this study was lower than that reported in other studies. Among many others, Tostain et al. (2006) from Benin and Cao et al. (2021) from China found a total of 124 and 96 alleles, respectively, on 156 and 112 accessions of *Dioscorea* species using 17 and 23 SSR markers. A considerably higher number of total alleles (256) was also reported by Arnau et al. (2017) in France. The high discrepancy between our results and those of earlier findings may be due to the use of different genotypes as well as the difference in the number of accessions and primers used.

In our study, the markers produced on average 7.2 alleles, with a range of 5 to 11 alleles per locus, indicating the presence of different ploidy levels in the study accessions. The mean number of alleles per locus was higher than those in previous studies by Wu et al. (2019) (Na = 1.81) and Cao et al. (2021) (Na = 4) in Chinese yam, while it was lower than that reported by Loko et al. (2017) for Guinea yam (Na = 8.69) and Korsa et al. (2022) in Ethiopian yam (Na = 9.2). The results showed that SSR markers used in this study were able to effectively capture the genetic variation of *Dioscorea* species.

Gene diversity or expected heterozygosity obtained here was found to be moderate, 0.38 on average, ranging from 0.23 to 0.48 per locus. The mean gene diversity found here was higher than that in Bekele (2014) (He = 0.32) reported for Ethiopian yams using SSR markers. It was, however, lower than that reported by Tostain et al. (2007) in Benin (He = 0.58), Siqueira et al. (2012) in Brazil (He = 0.69), Mengesha et al. (2013a) in Ethiopia (He = 0.64), Otoo et al. (2015) (He = 0.92) in Ghana, and Arnau et al. (2017) in France (He = 0.66). The variation in the level of gene diversity might be due to the difference in the number and types of yam species used in the different studies.

The loci showed differences between expected and observed heterozygosity, indicating the presence of excess heterozygosity (Table 2). This in turn reflects the existence of considerable genetic diversity among the study accessions. The reason for this variability is that yam is a crop that is propagated vegetatively and usually maintains a high heterozygosity level (Scarcelli et al. 2006a, 2006b). According to Scarcelli, Tostain, Mariac, et al. (2006), domestication practice involves plants produced by sexual reproduction of wild yams, and through this practice, sexual reproduction contributes to the evolutionary dynamics of yam. Likewise, in Ethiopia, farmers usually collect tubers of feral or possibly wild types from wild locations, plant them in their gardens, and through the course of clonal propagation, they select suitable ones (Hildebrand 2003; Worojie et al. 2021b). From the facts presented here, it can be concluded that the observed genetic divergence in Ethiopian yams may be due to the practice of domestication and subsequent clonal propagation and selection by farmers. Similar results have been reported in Ethiopia and across the world on yam (Obidiegwu et al. 2009; Mengesha et al. 2013a, 2013b; Loko et al. 2017) and other crops like *Plectranthus edulis* (Gadissa et al. 2018) and Cassava (Tovar et al. 2015).

The PIC values varied from 0.18 to 0.36, with an average of 0.30. Markers with PIC values between 0.5 and 0.25 are reasonably informative (Botstein et al. 1980). About 80% of the markers used in our study showed a PIC between 0.5 and 0.25, indicating that they are informative enough and have the required properties to be used in the diversity study of Ethiopian yams. Yet, due to the fact that many of our plant materials have failed to produce good amplification products, more work may be needed to design new yam primers. This also provides a clue to have recourse to more powerful markers such as SNPs in the future genetic diversity study of Ethiopian yams. This result is in agreement with the average PIC value reported in Ethiopia (Mulualem et al. 2018), but much lower than the values (PIC = 0.87) reported by Korsa et al. (2022). The current study presented a lower level of PIC values when compared with similar studies in other countries. For example, in Brazil, Siqueira et al. (2012) reported PIC value ranges of 0.57 to 0.77, with an average of 0.66 on 36 *D. alata* accessions using 12 loci. Higher mean PIC values were also reported from West Africa by Obidiegwu et al. (2009) on *D. cayenensis* /*rotundata* accessions with 15 SSR loci (PIC = 0.65) and Obidiegwu et al. (2010) on *D. alata* accessions with 13 loci (PIC = 0.65).

### 4.2 Genetic Diversity Among Populations

The amount of genetic diversity parameters that were found in this study is a reflection of the richness of the populations and the variability among the study accessions. The mean number of alleles per locus among populations varied from 4.9 in SHK to 7 in BMG (Table 3), with the grand mean of all populations being 6.13. The mean number of alleles per locus observed here was much higher than the 3.21 alleles reported in four populations of 72 *D. alata* accessions from Brazil (Siqueira et al. 2014), or the 1.93 alleles found in seven populations of 33 yam accessions from Ethiopia (Mulualem et al. 2018). The mean number of effective alleles (Ne = 1.51), expected heterozygosity (He = 0.29) and Shannon index (I = 0.43) obtained in this study were found to be moderate. These results are lower than the values recorded in previous studies by Tostain et al. (2007) in Benin (He = 0.52, I = 0.5), Arnau et al. (2017) in France (I = 0.66), Olu‐Olusegun et al. (2018) in Japan (He = 0.68) and Korsa et al. (2022) in Ethiopia (Ne = 4.45, He = 0.73) for yam populations using SSR markers. The Shannon diversity index obtained here (0.43), however, was higher than the mean values (0.34) obtained by Siqueira et al. (2014) in a genetic diversity study of 72 accessions representing four yam populations from Brazil. The variation in genetic diversity parameters could be due to the difference in the number of samples per population, as well as the difference in the type and context of genotypes used in different studies.

The populations of BMG resulted in higher values for the entire genetic diversity parameters assessed (Na = 7, Ne = 1.64, He = 0.34, I = 0.51 and PPL = 97.22), which were higher than their respective grand means. This could be due to the larger size of the population and wider genetic basis of the BMG populations. The observed genetic divergence could also partly be due to the practice of domestication by the local farmers, which is still actively ongoing in areas where BMG populations were collected (Hildebrand 2003; Mengesha et al. 2013a, 2013b; Worojie et al. 2021a, 2021b). The amount of genetic diversity within the populations of GEO was also equivalent to that of BMG populations. This may be due to the fact that the GEO population contains a noticeably high number of total alleles (65) in spite of its small sample size (Table 3). Nonetheless, Salazar et al. (2017) reported that the use of few samples as one population may lead to a deficit of alleles within that population. This could explain the differences found in genetic patterns of populations from Sheka.

Differences in the number of private alleles were observed among the study populations. In this regard, only BMG populations bear five private alleles, showing the existence of a unique genetic makeup within these populations. As pointed out by Slatkin (1985), at a certain level of independent evolution of gene pools, private alleles indicate unique genetic makeup and allow the preservation of private alleles at a population level. Future breeding programs could benefit from the presence of private alleles as a source of important traits. This result agreed with earlier findings, where the existence of private alleles in one population that were not present in others was reported on sorghum (Casa et al. 2005), yam (Siqueira et al. 2014; Korsa et al. 2022) and corn (Warburton et al. 2008; Salazar et al. 2017). The existence of private alleles in BMG populations reflects the presence of distinct genotypes that could be exploited in future yam improvement programs.

The percentage of polymorphic loci (PPL) per population ranged from 68.06% to 97.22%, with an average of 85.2% (Table 3). The level of polymorphic loci among populations obtained in this study is considerably high. This suggests that there is a high level of genetic variability among yam accessions in Ethiopia. Therefore, it can be utilized in a program intended to enhance the crop's genetic diversity. The mean PPL values obtained here are within the range of the previous studies. Otoo et al. (2015) and Mulualem et al. (2018) reported lower PPL values (53 and 58.6%) from Ghana and Ethiopia, while Siqueira et al. (2014) and Korsa et al. (2022) obtained higher values (92 and 98%) among the Brazilian and Ethiopian yams. Yet, much lower values of PPL were reported on maize and cowpea by earlier studies (Fatokun et al. 2018; Nelimor et al. 2020).

### 4.3 Genetic Relationship Among Populations

Analysis of molecular variance (AMOVA) showed the existence of low genetic differentiation among populations of distinct geographic origins. Most of the total variation (92%) was due to the high genetic variability observed among individuals within populations. In accordance with our result, a lower level of among‐population genetic variation was reported for yam across the world, including 2% in Japan (Olu‐Olusegun et al. 2018), 9% in Ethiopia (Korsa et al. 2022) and 12% in Kenya (Muthamia et al. 2013). Similarly, Gadissa et al. (2018) and Tolera et al. (2021) found a lower level of genetic variation among populations in *P. edulis* and anchote accessions from Ethiopia. On the other hand, Arnau et al. (2017) found the highest among‐population genetic variation (40.9%) for yam populations in France.

The level of genetic variation obtained in this study suggests that the genetic diversity is less affected by their geographic origin. The cluster study's visualization of genetic relationships showed that accessions were grouped sporadically without a distinct pattern of geographic origin. A similar result is also evident in a scatter plot from PCoA, in which scattering was not according to the geographic origin of accessions. Our result is in agreement with those reported for yam in Ethiopia through SSR markers (Bekele 2014; Mengesha et al. 2013a; Mulualem et al. 2018; Korsa et al. 2022). Similar results were observed worldwide on yam (Mignouna and Dansi 2003; Bhattacharjee et al. 2020; Agre et al. 2021), and other crops such as cowpea (Fatokun et al. 2018) and maize (Nelimor et al. 2020). In each of these studies, analysis of molecular clustering has failed to show any clear association with geographic origin. Our result, however, was not in accord with the findings of Tostain et al. (2007), who established a clear relationship between molecular clustering and geographic origins on the yam accessions in Benin.

Analysis of molecular clustering provided additional insight into the genetic structure among and within populations, in which the population structure bar plot clearly demonstrated a high level of gene flow between regions of Ethiopia. This gene flow reflects the presence of a wide‐ranging seed exchange networks for yam in Ethiopia, thereby maximizing the genetic variability among individuals within the population. As it is clear from this study that accessions from the BMG region were grouped together with the GEO area that is thousands of kilometers away from the region. Exchange of landraces among farmers is thus not confined to close neighbors, but extends to distant regions. Several other studies on anchote (Tolera et al. 2021), rosemary (Zigene et al. 2021), and yam (Tostain et al. 2007; Siqueira et al. 2014; Loko et al. 2017; Bhattacharjee et al. 2020; Agre et al. 2021) showed the existence of a high inter‐region seed diffusion networks, with the exchange extending to long distances. Most of these studies reported the presence of a high level of gene flow between regions without a clear pattern of geographical differentiation.

## 5 Conclusion

In this study, 10 SSR primers were used to analyze the genetic diversity of 31 yam accessions. The use of SSR markers in this study resulted in an efficient and reasonably informative genetic diversity study of yams. The pattern of diversity across the collection sites showed that Bench‐Maji and Gambella populations showed higher values for all the genetic diversity parameters assessed. Despite its small sample size, the Gedeo and Oromia populations also exhibit a high amount of genetic diversity. These areas could thus be considered as potential areas for diverse yam species. However, the populations of BMG possess private alleles, which strongly suggests that genotypes from BMG may serve as a source of important traits for broadening the genetic base of Ethiopian yams.

In the cluster analysis, no clear pattern of geographic variation was detected among accessions from different geographic origins. AMOVA's partitioning of genetic variation supports this idea, demonstrating very low variability among populations, with the variability largely distributed within the population. This entire pattern suggests that yam growers in Ethiopia might have followed seed diffusion systems not limited to neighboring areas. Future improvement and conservation efforts on yam should thus take the on‐farm diversity into account.

In conclusion, the results of this finding have important implications for the collection and conservation of yam landraces in Ethiopia. Yet, the study is not exhaustive. We also managed to consider a few yam genotypes, the fact that 105 of the 136 yam accessions have failed to produce better amplification products. Additional genetic diversity studies' involving a larger sample size and more powerful markers are of paramount importance to detect the full extent of yam genetic diversity in Ethiopia.

## Author Contributions

**Gerezgiher Mekonen Gebrhud:** data curation (equal), formal analysis (equal), investigation (equal), writing – original draft (equal). **Hewan Demissie Degu:** conceptualization (equal), funding acquisition (equal), supervision (equal), writing – review and editing (equal). **Eleni Shiferaw Tessema:** conceptualization (equal), resources (equal), writing – review and editing (equal). **Tsegaye Babege Worojie:** conceptualization (equal), funding acquisition (equal), writing – review and editing (equal).

## Conflicts of Interest

The authors declare no conflicts of interest.

## Supporting information


Data S1.

## Data Availability

All the required data are uploaded as Supporting Information.

## Footnotes

1
*Note:* The bold values indicate the average of each parameters for the 10 SSR markers.

2 Abbreviations: GD/He, gene diversity or expected heterozygosity; Ho, Observed heterozygosity per locus; MAF, maximum allelic frequency; Na, average number of alleles per locus; Ne, number of effective alleles per locus; PIC, polymorphic information content.
