# Supplementary material for: Genetic Variability and Population Structure of Ethiopian Yams (Dioscorea spp.) Based on SSR Markers
Source: Ecol Evol. 2025 Jun 5;15(6):e71562. doi: 10.1002/ece3.71562 (PMC12141759; doi:10.1002/ece3.71562)

Supplementary material

ESM 1. Scientific and local names, collection area and geographic origins of the 31 accessions

| Accession codes | | Local names | Scientific names | Geographic origin and coordinates | | | | Altitude(masl) |
| --- | --- | --- | --- | --- | --- | --- | --- | --- |
|  |  |  |  | Region | Zone | Latitude | Longitude |  |
| 1 | ATS005 | - | *D. alata* | Gambella | MAJ | 07-13-14N | 35-16-37E | 1216 |
| 2 | TY107 | - |  | Oromia | - | 09-01-57N | 36-39-50 | 1919 |
| 3 | ATS056 | *-* |  | SNNPRS | BMJ | 06-48-44N | 35-15-17E | 1265 |
| 4 | WNA127 | - | *D. bulbifera* |  | GE | 06-10-40N | 038-15-51E | 2083 |
| 5 | WNA161 | - |  |  |  | 18-06-13N | 038-16-17E | 1681 |
| 6 | WNA178 | - | *D. alata* | Oromia | BO | 24-06-16N | 038-17-02E | 1442 |
| 7 | ATS014 | - |  | Gambella | MAJ | 07-11-59N | 35-13-50E | 1006 |
| 8 | WNA123 | - |  | SNNPRS | GE | 06-10-31N | 038-14-00E | 1964 |
| 9 | BMJ020 | *Shapinsin* | *D. cayenensis* complex |  | BMJ | 06-49-47N | 35-29.17-20E | 1380 |
| 10 | BMJ004 | *Karka-Kachi** |  |  |  | 07-2-43.5N | 35-31-18.0E | 1677 |
| 11 | BMJ002 |  |  |  |  | 07-2-34.05N | 35-31-0.05E | 1696 |
| 12 | BMJ009 | *Torbay* |  |  |  | 07-2.18-30N | 35-31.1029E | 1645 |
| 13 | BMJ022b | *Tsid boy* |  |  |  | 07-3-58.84N | 35-40-02.59E | 1855 |
| 14 | BMJ014a | *Kachi-Tsano* |  |  |  | 07-2-21.16N | 35-31-10.35E | 1696 |
| 15 | BMJ023 | *Tsid boy* |  |  |  | 07-4-10.4N | 35-39-8.25E | 1810 |
| 16 | SHK007 | *Konkay* |  |  | SH | 07-8-39.20N | 35-24-12.89E | 1152 |
| 17 | SHK004 | *Kachi ga’nseb* |  |  |  | 07-12-44N | 35-22-24.06E | 1293 |
| 18 | SHK009b | *Kachi tsaa’nseb* |  |  |  | 07-8-44.88N | 35-24-17.00E | 1162 |
| 19 | BMJ008 | *Beri* |  |  | BMJ | 07-2-18.30N | 35-31-10.29E | 1645 |
| 20 | BMJ019 | *Dal boy* |  |  |  | 06-49-51N | 35-29-10.23E | 1399 |
| 21 | BMJ021 | *Tsid boy* |  |  |  | 06-29-47N | 35-29-17.00E | 1380 |
| 22 | BMJ013 | *Dizzu kachi* |  |  |  | 07-2-34.05N | 35-31-10.65E | 1296 |
| 23 | BMJ034c | *Karka-Kachi*** |  |  |  | 07-01-09.5N | 35-33-45.2E | 1300 |
| 24 | BMJ030 | *Tsid boy* |  |  |  | 07-3-38.73N | 35-38-38.15E | 1706 |
| 25 | BMJ032 | *Banda boy* |  |  |  | 06-49-51N | 35-29-09.26E | 1390 |
| 26 | SHK003 | *Kachi ga’nseb* |  |  | SH | 07-12-46N | 35-22-27.51E | 1305 |
| 27 | BMJ029 | *Tsid boy* |  |  | BMJ | 06-49-46N | 35-29-15.59E | 1400 |
| 28 | BMJ028a | *Shamut* |  |  |  | 06-49-46N | 35-29-15.59E | 1400 |
| 29 | BMJ033b |  |  |  |  | 06-49-54N | 35-21-12.34E | 1402 |
| 30 | SHK009a | *Kachi tsaa’nseb* |  |  | SH | 07-8-44.88N | 35-24-17.20E | 1162 |
| 31 | BMJ033a | *Shamut* |  |  | BMJ | 06-49-54N | 35-29-12.34E | 1402 |

*= wild transplant, ** = wild; MAJ = Majang, BMJ = Bench-Maji, GE = Gedio, BO = Borena, SH = Sheka NB: Bench-Maji is split into two administrative Zones; Bench-Sheko and Southwest Omo. Thus, BMJ accessions now represented Bench-Sheko. Accordingly, BMJ and SH populations now represented the newly established region, known as Southwest Ethiopia Region.

**ESM 2.** Original Map of Collection Area Constructed using Geographic Coordinates and Elevation Data Gathered from ach Collection Sites using GPS


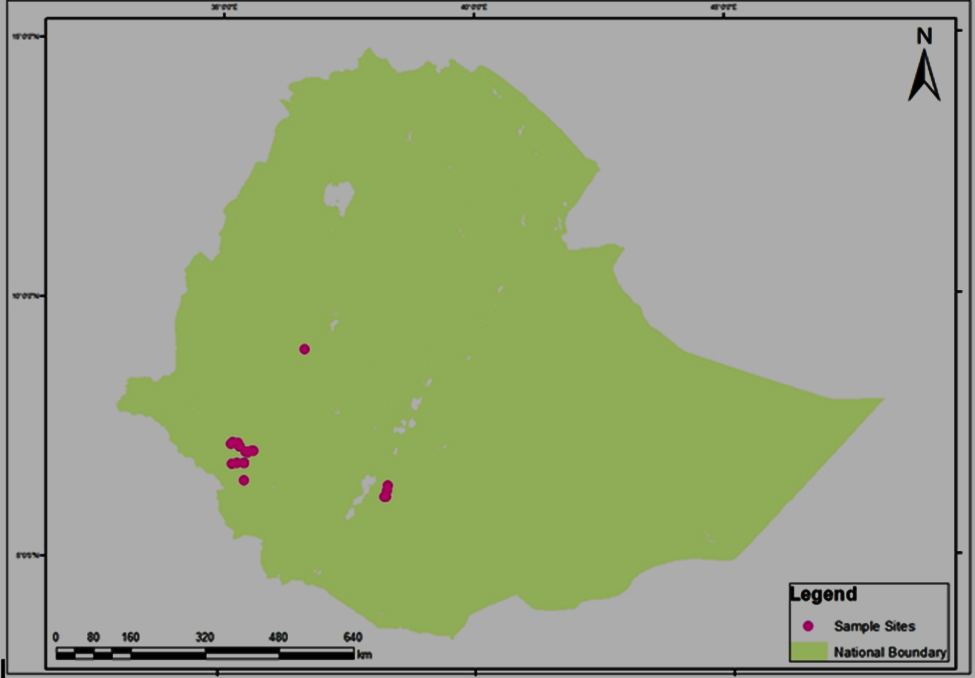

Supplement: Supplementary file 1 — Data S1. [file ECE3-15-e71562-s001.docx]
